# Post-traumatic Sacral Epidermoid Cyst Masquerading as Chordoma on Imaging: A Case Report

**DOI:** 10.31729/jnma.4268

**Published:** 2020-03-31

**Authors:** Subash Phuyal, Anuj Prabhakar, Parthiban Balasundaram, Shailesh Gaikwad

**Affiliations:** 1 Department of Neuroimaging and Interventional Neuroradiology, AIIMS, New Delhi, India

**Keywords:** *epidermoid cyst*, *intradural-extramedullary spinal cord neoplasms*, *restricted diffusion*, *spinal canal*

## Abstract

Intradural spinal epidermoid cysts are rare, benign lesions either acquired from trauma, surgery, lumbar puncture or arise as congenital lesions, particularly associated with spinal dysraphism. Epidermoid cyst arising from the spine with expansile destruction of vertebrae has not been reported yet in the literature. We report a case of 36-years male presented with history of fall 8 years back with progressive symptoms of lower back pain, weakness of left lower limb and bladder/bowel incontinence. Computed tomography revealed large lytic expansile, midline sacral vertebral lesion with soft tissue component and multiple calcific foci. Magnetic resonance imaging demonstrated large non-enhancing heterogeneous mass showing restricted diffusion on diffusion weighted images. The patient underwent biopsy confirming the diagnosis of an epidermoid cyst. The possibility of an epidermoid tumor should be kept in the differential diagnosis in patients presenting with posttraumatic sacral mass.

## INTRODUCTION

Epidermoid cysts are benign, slow-growing lesions and may often reach an enormous size without producing neurological symptoms. In the spine, the common locations of epidermoid tumors are intradural spinal canal and prevertebral. Intradural spinal epidermoid tumors are rare, comprising less than 1% of tumors involving the spine.^1^ Spinal epidermoids are typically found in the lumbosacral region but can be found in other locations as well.^2^ Patients usually present with pain as well as neurologic dysfunction that may include, muscle weakness and atrophy, sensory disturbances, and loss of sphincter control.^3^

## CASE REPORT

A 36-years male, presented with low back pain and leftsided sciatica. Patient had experience occasional mild low back pain for several years which was markedly worsened in the last 4 months. He also had progressive urinary urgency and bowel incontinence for same duration.

On clinical examination, the patient had 2/5 power on left foot dorsiflexion and plantar flexion, 1/5 power in left hip flexion and knee extension, while no weakness was seen in the right lower limb. The sensation was grossly intact and his reflexes were normal without hyperreflexia.

**Figure 1:**
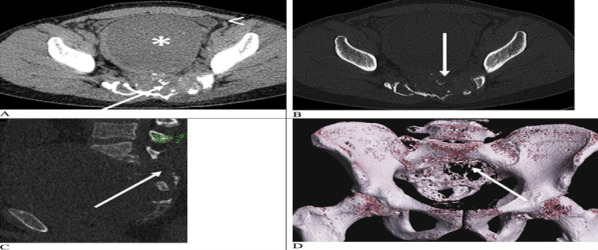
Axial soft tissue and bone window computed tomography of the pelvis (a and b) shows large lytic expansile midline lesion, destruction of sacrum with bilateral neural foraminal and sacral canal components. Multiple calcific foci are seen within the mass (white arrows). The lesion has large, predominantly cystic presacral component (* in a), which is seen displacing urinary bladder anteriorly (arrowhead in a). Sagittal bone window computed tomography (c) and volume-rendered images (d) shows lytic destruction of the sacrum (white arrows in c and d).

CT of the lumbosacral region showed lytic expansile midline lesion causing destruction of sacrumwithlarge presacral component, bilateral neural foraminal and sacral canal components (Figure 1). Based on history and CT imaging, the differential diagnosis included chordoma, epidermoid tumor, schwannoma and chondrosarcoma.

**Figure 2:**
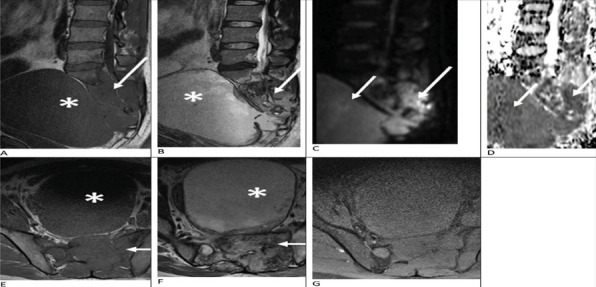
Sagittal T1 and T2 weighted images of the pelvis (a and b) shows heterogeneous mass (isointense signal on T1-w and hyperintense signal on T2-w) involving sacral vertebra and adjacent spinal canal (white arrows in a and b), with large presacral component (* in a and b). Sagittal diffusion-weighted images and apparent diffusion coefficient map (c and d) shows diffusion restriction evidenced by hyperintense signal (white arrows in c) and hypointense signal (white arrows in d). Axial T1 and T2 weighted images(e and f) shows heterogeneous mass involving the sacrum (white arrows in e and f) with presacral component (* in e and f). Post-contrast fat-saturated axial T1 weighted image (g) shows no obvious enhancement.

Subsequent magnetic resonance imaging (MRI) demonstrated heterogeneous non-enhancing mass involving presacral region, sacral vertebra and adjacent spinal canal (Figure 2). Diffusion-weighted imaging and apparent diffusion coefficient map showed diffusion restriction of the lesion. MRI findings of diffusion restriction without any enhancement favored the diagnosis of an epidermoid cyst. Other differential diagnoses such as chordoma and chondrosarcoma were ruled out, as there was no enhancement, especially in the diffusion restricting regions. A trucut biopsy was performed which revealed an epidermoid tumor.

## DISCUSSION

Intradural spinal epidermoid cysts are rare, benign lesions that are either acquired from trauma, surgery, lumbar puncture or arise as congenital lesions, particularly associated with spinal dysraphism. Since the clinical symptoms of spinal epidermoid tumors are variable and non-specific with slow progression, the diagnosis may be delayed. Spinal epidermoid tumors are difficult to diagnose on clinical symptoms alone. Plain radiographs usually show normal or non-specific findings, especially in acquired spinal epidermoid tumors.

A specific histologic feature of an epidermoid cyst is a layer of stratified squamous keratinizing epithelium surrounded by an outer layer of collagenous tissue, with the absence of skin adnexa. The desquamation of keratin from the epithelial lining creates numerous cholesterol crystals.^4^ The etiologies of spinal epidermoid tumors are thought to be both congenital and acquired. Acquired epidermoid were mostly reported in the region of the cauda equina because lumbar punctures are usually performed around the level of the iliac crests.^5,6^ To the authors' best knowledge, there has never been any report describing acquired spinal epidermoid tumors involving the vertebral body after trauma and that too with a large presacral component. In our case, skin epithelium might have got implanted in the sacrum and pre-sacral region, secondary to trauma. Surgical treatment is required when the patients develop neurological symptoms. Complete excision of epidermoid is the surgical treatment of choice. However, complete resection of the encapsulated tumor is difficult in all cases, because the epidermoid capsules are very thin and the tumor often adheres to the arachnoid membrane, spinal cord, or nerve roots. Thereby, subtotal resection is commonly performed.^7,8,9,10^

Epidermoid cysts have specific imaging findings, especially in MRI that can help in the diagnosis. The lesions follow fluid-like signals in T1 and T2 weighted images. Lesions show diffusion restriction in diffusionweighted images, as in our case, which is commonly due to the orientation of the keratin layers which restrict the free movement of water. They show minimal to no enhancement, especially the diffusion restricting regions, contrasting with malignant tumors.

## Consent:

**JNMA Case Report Consent Form** was signed by the patient and the original article is attached with the patient's chart.

## Conflict of Interest

**None.**
